# Lysosomal Hydrolase Cathepsin D Non-proteolytically Modulates Dendritic Morphology in *Drosophila*

**DOI:** 10.1007/s12264-020-00479-6

**Published:** 2020-03-14

**Authors:** Ting Zhang, Daxiao Cheng, Cunjin Wu, Xingyue Wang, Qiang Ke, Huifang Lou, Liya Zhu, Xiao-Dong Wang, Shumin Duan, Yi-Jun Liu

**Affiliations:** 1 Department of Neurobiology, Key Laboratory of Medical Neurobiology of the Ministry of Health of China, Zhejiang Province Key Laboratory of Neurobiology, Zhejiang University School of Medicine, Hangzhou, 310058 China; 2 Department of Diagnostics, Hangzhou Normal University Medical School, Hangzhou, 311121 China; 3 Department of Laboratory Medicine, Hangzhou Normal University Affiliated Hospital, Hangzhou, 310015 China; 4 Department of Psychiatry, Sir Run Run Shaw Hospital, Zhejiang University School of Medicine, Hangzhou, 310016 China; 5 Mental Health Center, Zhejiang University School of Medicine, Hangzhou, 310058 China

**Keywords:** Cathepsin D, Dendritic arborization, Sensory neuron, Mushroom body, Actin, *Drosophila*

## Abstract

The main lysosomal protease cathepsin D (cathD) is essential for maintaining tissue homeostasis *via* its degradative function, and its loss leads to ceroid accumulation in the mammalian nervous system, which results in progressive neurodegeneration. Increasing evidence implies non-proteolytic roles of cathD in regulating various biological processes such as apoptosis, cell proliferation, and migration. Along these lines, we here showed that cathD is required for modulating dendritic architecture in the nervous system independent of its traditional degradative function. Upon *cathD* depletion, class I and class III arborization (da) neurons in *Drosophila* larvae exhibited aberrant dendritic morphology, including over-branching, aberrant turning, and elongation defects. Re-introduction of wild-type cathD or its proteolytically-inactive mutant dramatically abolished these morphological defects. Moreover, cathD knockdown also led to dendritic defects in the adult mushroom bodies, suggesting that cathD-mediated processes are required in both the peripheral and central nervous systems. Taken together, our results demonstrate a critical role of cathD in shaping dendritic architecture independent of its proteolytic function.

**Electronic supplementary material:**

The online version of this article (10.1007/s12264-020-00479-6) contains supplementary material, which is available to authorized users.

## Introduction

Endosome/lysosomes are key organelles in eukaryotic cells, containing a wide variety of hydrolases responsible for the turnover of macromolecules. Among the lysosomal hydrolases, cathepsin D (cathD) is the main acidic hydrolase responsible for nonspecific protein degradation [1]. Although other lysosomal hydrolases may replace cathD to mediate proteolysis, cathD-knockout mice exhibit serious and systemic abnormalities before death around postnatal day 26 [2], suggesting a unique role of cathD that differs from other lysosomal proteases.

Beyond its traditional role in proteolysis, increasing evidence indicates that cathD has distinct functions independent of its proteolytic activity [3]. For instance, both mature cathD and its proteolytically-inactive mutant (cathD^D231N^) promote apoptosis, cell proliferation, and migration [4–6]. Intriguingly, cathD also exists outside endosome/lysosomes, where the pH is far beyond the optimal range for its canonical proteolytic action [7–9]. These findings suggest that cathD exerts non-proteolytic activities that control normal cellular processes.

CathD is required for maintaining homeostasis in the central nervous system (CNS). Reduction of the protein level or the proteolytic activity of cathD leads to mental and motor deterioration in humans. Similarly, cathD-deficient mice exhibit rapidly progressive neurodegeneration [10, 11] with symptoms including seizures, ataxia, and visual disturbances. On the one hand, the neurological disorders induced by *cathD* depletion are accompanied by the lysosomal storage of undigested materials [10, 12], suggesting that cathD modulates neuronal metabolism *via* its proteolytic action. On the other hand, as cathD may non-proteolytically modulate various cellular processes [2, 5, 13], it is possible that it also regulates CNS homeostasis *via* non-proteolytic actions.

In contrast to other species, *Drosophila* has a normal life-span upon *cathD* depletion [12], making it available for investigation at different life stages. In this study, we used larval and adult *Drosophila* to investigate the regulatory role of cathD in neurodevelopment.

## Material and Methods

### *Drosophila* Stocks

All flies were raised on standard meal medium in 60% humidity and with a 12-h light/12-h dark cycle at 25°C. *w1118*, *cathD*^*1*^, *UAS-cathD*^*wt*^, *UAS-cathD*^*D231N*^, *UAS-cathD*^*RNAi*^, *UAS-actin*^*G15S*^, *UAS-acitn*^*R62D*^, and *OK107-Gal4* were kindly provided by Prof. Margaret S. Ho. *2-21-Gal4*, *UAS-mCD8-GFP*, and *19-12-Gal4* were gifts from Prof. Zhiqiang Yan. *UAS-rab7*^*RNAi*^ (V40338) and *UAS-ssh*^*RNAi*^ (V107998) were purchased from the Vienna Drosophila Resource Center. For observation of cathD-depleted class I and III neurons, *19-12-Gal4*; *UAS-mCD8-GFP* and *2-21-Gal4, UAS-mCD8-GFP* were each crossed into the *cathD*^*1*^ background. To visualize cathD-knockdown mushroom bodies (MBs), *OK107-Gal4; UAS-mCD8-GFP* was crossed transiently with *UAS-cathD*^*RNAi*^.

### *In Vivo* Imaging of Larval Sensory Neurons

After anesthesia with diethyl ether for 5–10 min, individual larvae at 120 h after egg laying were placed on a glass slide and covered with Halocarbon oil 700 (Sigma, St. Louis, MO). Then a coverslip with a plastic spacer was gently placed on top of the larva for immobilization. To visualize class I and class III da neurons, image stacks from the dorsal region of segment A3 at a step size of 1 μm were acquired using a confocal microscope (FV1200, Olympus, Tokyo, Japan) with a 20×, 0.75 NA objective. These stacks were subsequently projected to final 2-D images avoiding loss of dendritic branches in optical sectioning. For image quantification, the length of a dendrite was measured using the NeuronJ plugin of ImageJ (ImageJ 1.52p, NIH, Bethesda, MD), and dendritic branching was determined using the Sholl Analysis plugin of ImageJ. Dendritic angles were measured using the ‘Angle’ tool in ImageJ. Aberrant turning of dendrites was defined and categorized as clear turning (angles <90°) during dendrite elongation.

### Dissection and Imaging of Adult MBs

The brains of flies with GFP-labeled MBs were dissected from fixed adults 3–5 days after eclosion and further prepared for imaging as previously described [14, 15]. Image stacks of MBs were acquired at a step size of 0.47 μm using a confocal microscope (Olympus, FV1200) equipped with 60×, 1.2 NA and 20×, 0.75 NA objectives. All image stacks were de-convolved with Huygens Professional version 19.04 (Scientific Volume Imaging, Hilversum, The Netherlands), and then reconstructed using Imaris software (Bitplane, South Windsor, CT). Volumes of Kenyon cell (KC) somata and their calyces were calculated from area measurements using ImageJ.

### Statistical Analysis

Data were analyzed using GraphPad Prism 8.0 (GraphPad Software Inc., San Diego, CA). Statistical methods and results are summarized in Table S1. Data are presented as the mean ± SEM. *P* < 0.05 was considered to be statistically significant.

## Results

### CathD Regulates Dendritic Architecture in Sensory Neurons

**Fig. 1:**
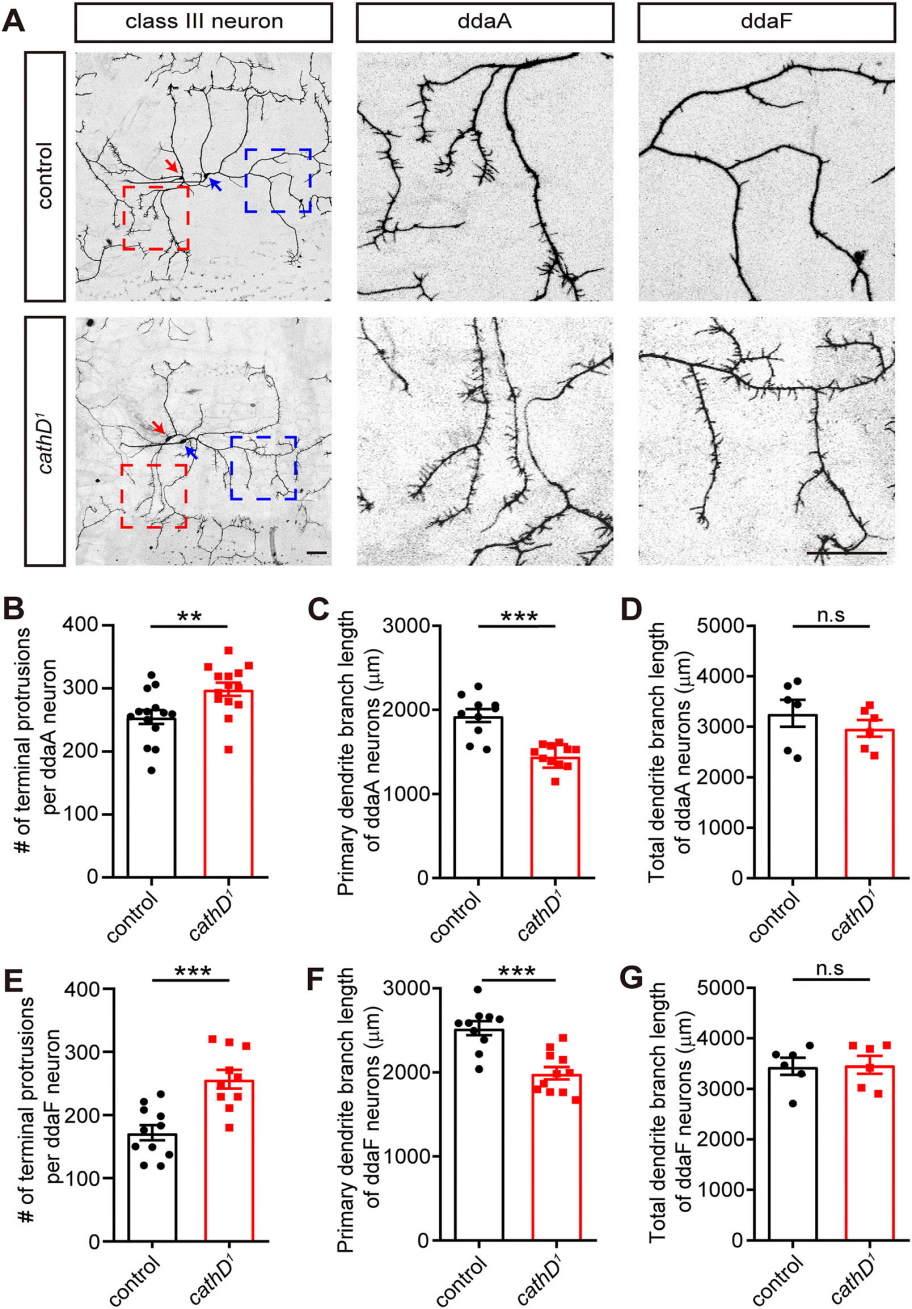
Loss of cathD leads to morphological defects in ddaA and ddaF class III da neurons. **A** Representative images of wild-type (control) and cathD-deficient (*cathD*^*1*^) neurons labeled with *19-12-Gal4; UAS-mCD8-GFP* (red arrows, ddaA neurons; blue arrows, ddaF neurons; boxed regions enlarged in right panels; scale bars, 50 μm). **B** Numbers of terminal protrusions per ddaA neuron. **C**, **D** Quantification of primary dendrite branch length (**C**) and total dendrite branch length (**D**) of ddaA neurons. **E**–**G** Numbers of terminal protrusions (**E**), primary dendrite branch length (**F**), and total dendrite branch length (**G**) of ddaF neurons. Data are the mean ± SEM; **P < 0.01, ***P < 0.001, unpaired Student’s *t* test.

To assess the potential role of cathD in dendritic morphogenesis, we first analyzed the effects of *cathD* depletion on class III da neurons, whose dendrites are characterized by short, filopodia-like protrusions emanating from primary branches [16, 17]. Driven by specific 19-12-Gal4, ddaA and ddaF class III da neurons were labeled with GFP in *cathD*-depleted (*cathD*^*1*^) larvae, allowing subsequent observation and assessment of dendritic morphology. Compared with controls, loss of cathD induced a complicated morphology featuring rich filopodia-like protrusions in both ddaF and ddaA neurons (Fig. 1A).

Detailed analysis showed that *cathD* depletion increased the number of terminal protrusions and decreased the primary branch length, without affecting the total dendritic length in ddaF neurons (Fig. 1E–G). Similar phenotypes were observed in ddaA neurons (Fig. 1B–D). These data suggest that cathD promotes dendritic elongation and controls dendritic branching in both ddaA and ddaF class III da neurons.

### CathD Non-proteolytically Controls Dendritic Architecture in ddaD Neurons

**Fig. 2:**
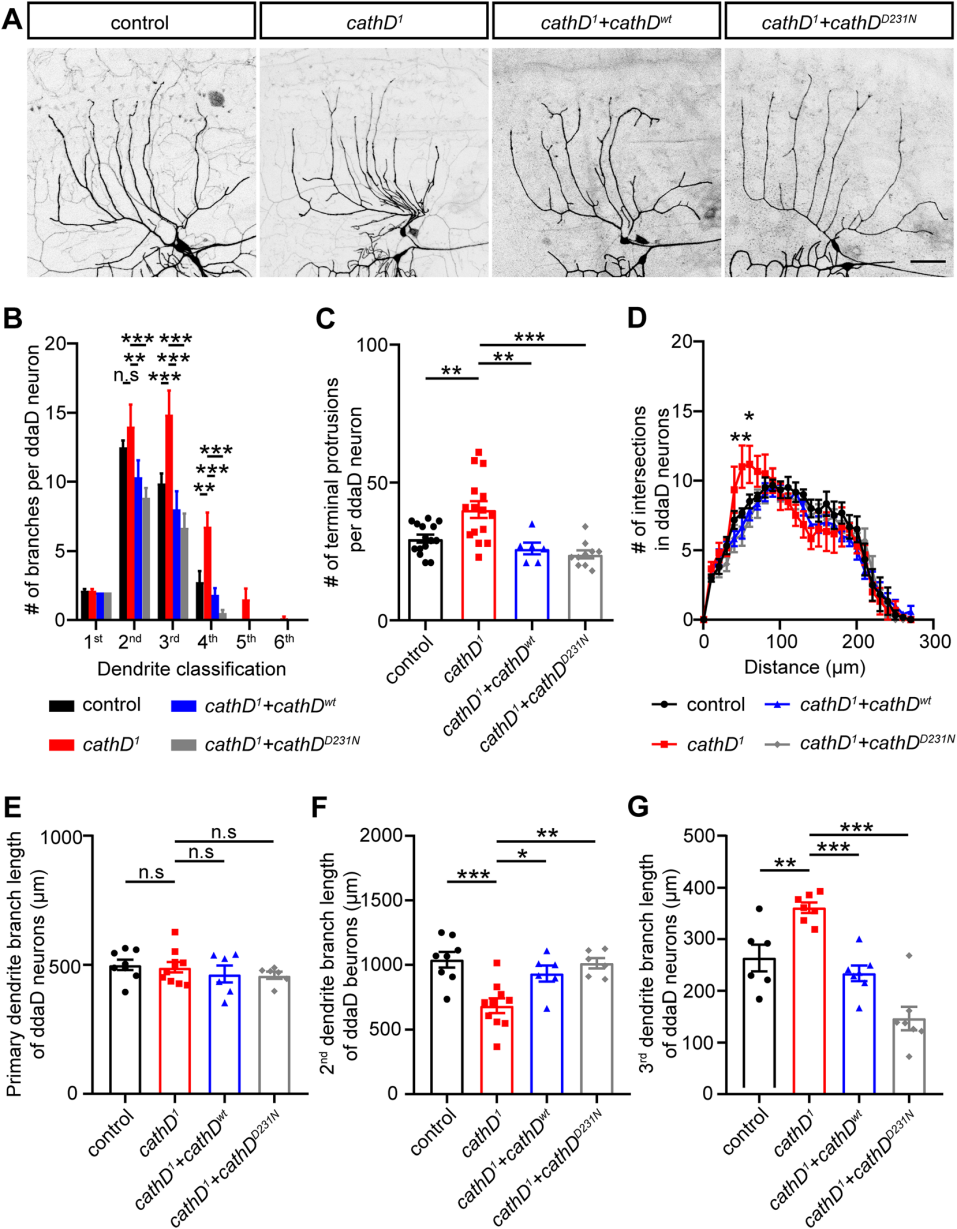
CathD non-proteolytically regulates the dendritic morphogenesis of ddaD neurons. **A** Representative images of *2-21-Gal4, UAS-mCD8-GFP*-labeled ddaD class I da neurons upon re-introduction of wild-type cathD (*cathD*^*wt*^) or proteolytically-inactive cathD (*cathD*^*D231N*^) in the cathD-deficient (*cathD*^*1*^) background (scale bar, 50 μm). **B** Quantification and distribution of dendritic branches of different orders, showing that expression of the wild-type (*cathD*^*wt*^) or proteolytically-inactive cathD (*cathD*^*D231N*^) reduces dendritic branching in the cathD-deficient (*cathD*^*1*^) background. **C** Quantification of terminal protrusions in ddaD neurons, showing that expression of the wild-type (*cathD*^*wt*^) or proteolytically-inactive cathD (*cathD*^*D231N*^) restores the over-branching phenotype in terminals with cathD-deficiency (*cathD*^*1*^). **D** Sholl analysis of dendrites of ddaD neurons with the indicated genotypes showing significant differences between control and cathD-deficient (*cathD*^*1*^) neurons in the number of dendritic intersections at 50–60 μm from the soma, which is abolished by re-introduction of wild-type (*cathD*^*wt*^) or proteolytically-inactive (*cathD*^*D231N*^) cathD. **E**–**G** Quantification of dendritic branch length in primary (**E**), secondary (**F**), and tertiary dendrites (**G**), showing the rescue effects of both wild-type (*cathD*^*wt*^) and proteolytically-inactive (*cathD*^*D231N*^) cathD in the cathD-deficient (*cathD*^*1*^) background. Data are shown as the mean ± SEM; *P < 0.05, **P < 0.01, ***P < 0.001, two-way ANOVA or one-way ANOVA with Tukey’s multiple comparisons test.

We next confirmed the regulatory role of cathD in ddaD class I da neurons that exhibit a simple branching pattern. Upon *cathD* depletion, the dendrites of ddaD neurons presented a more complicated morphology with more aberrant features, whereas reintroduction of wild-type or proteolytically-inactive cathD largely restored the normal phenotype in cathD-depleted ddaD neurons (Fig. 2A). Similar to the phenotypes in cathD-deficient class III da neurons, *cathD* depletion increased dendritic branching and terminal protrusions in ddaD class I neurons, and this was rescued by the re-introduction of wild-type (*cathD*^*wt*^) or proteolytically-inactive cathD (*cathD*^*D231N*^) (Fig. 2B, C). Sholl analysis revealed that *cathD* depletion increased the number of dendritic intersections proximal to the soma (Fig. 2D). Without affecting the length of primary dendritic branches (Fig. 2E), *cathD* depletion decreased the length of secondary dendrites, and increased the length of tertiary dendrites, both of which were restored by expressing cathD^wt^ or cathD^D231N^ (Fig. 2F, G). These results suggest a non-proteolytic role of cathD in regulating dendritic architecture.

**Fig. 3:**
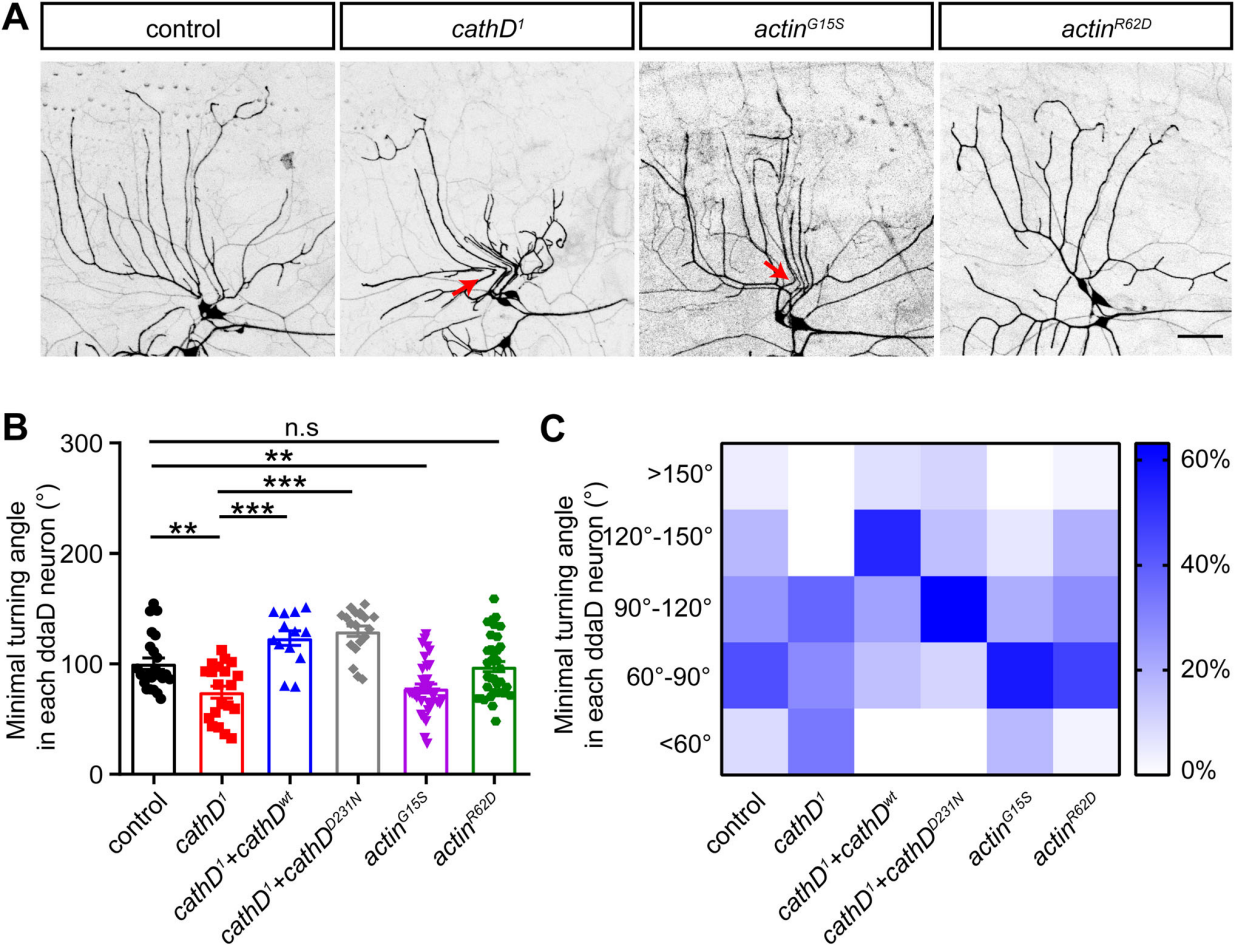
CathD modulates actin-mediated dendritic turning in ddaD neurons. **A** Representative images of *2-21-Gal4, UAS-mCD8-GFP*-labeled ddaD neurons, showing that cathD deficiency (*cathD*^*1*^) or actin^G15S^ overexpression (*actin*^*G15S*^) leads to aberrant turning in dendrites (arrows, acute angles in dendritic branches; scale bar, 50 μm). **B** Quantification of the minimal turning angle in each neuron, showing the expression of wild-type (*cathD*^*wt*^) or proteolytic-inactive (*cathD*^*D231N*^) cathD rescued acute turning in the cathD-deficient (*cathD*^*1*^) background. **C** Distribution heatmap of neuron ratios according to genotype and the minimal turning angle in dendritic branches. Data are shown as the mean ± SEM; **P < 0.01, ***P < 0.001, one-way ANOVA with Tukey’s multiple comparisons test.

We found that *cathD* depletion induced acute turning of dendrites in ddaD neurons (Fig. 3A). A similar phenotype also occurred upon overexpression of an actin mutant that favored polymerization (actin^G15S^), but was rarely seen upon overexpression of an actin mutant inhibiting actin assembly (actin^R62D^) [18–20], suggesting an over-stabilization state of the actin cytoskeleton in cathD-depleted ddaD neurons (Fig. 3A). Compared with control ddaD neurons, *cathD* depletion or expression of the actin^G15S^ mutant enhanced turning features in dendrites, while the expression of actin^R62D^ showed a less marked effect. Notably, re-introduction of wild-type or proteolytically-inactive cathD rescued the aberrant turning in ddaD neurons (Fig. 3B). In addition, *cathD* depletion and over-assembly by actin^G15S^ overexpression increased the percentage of dendrites with acute turns, which was rescued with both cathD^wt^ and cathD^D231N^ (Fig. 3C). These results suggest that cathD modulates dendritic architecture, especially dendritic branching and turning, independent of its proteolytic activity.

### CathD Shapes the Dendritic Architecture of ddaE Neurons Independent of Its Proteolytic Activity

**Fig. 4:**
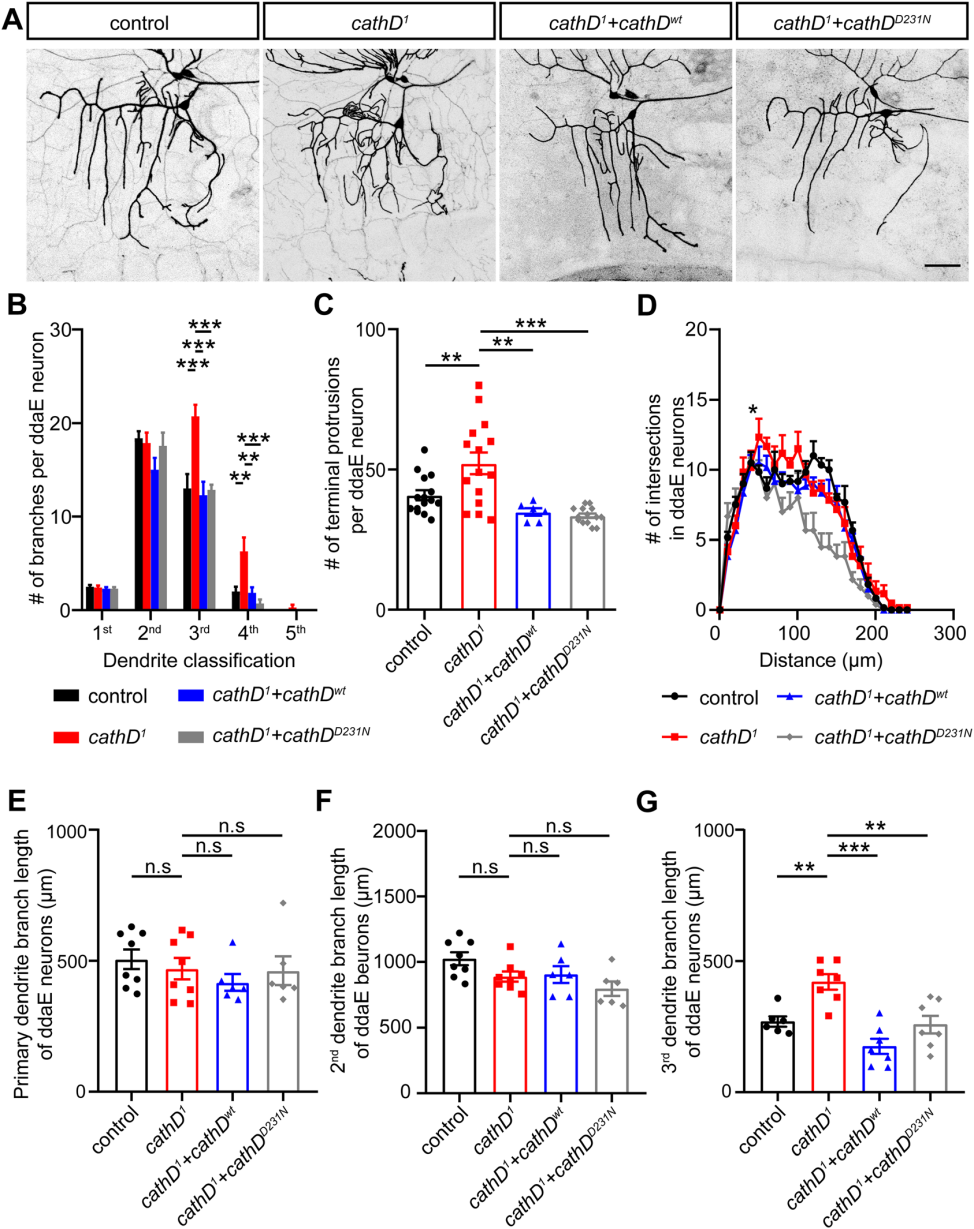
CathD non-proteolytically controls the dendritic morphogenesis of ddaE neurons. **A** Representative images of *2-21-Gal4, UAS-mCD8-GFP*-labeled ddaE class I da neurons in different genotype backgrounds. Compared with wild-type controls, re-introduction of wild-type cathD (*cathD*^*wt*^) or proteolytically-inactive cathD (*cathD*^*D231N*^) reduces the aberrant dendrites in cathD-deficient (*cathD*^*1*^) neurons (scale bar, 50 μm). **B** Quantification and distribution of dendrite branches of different orders in each ddaE neuron. **C** Quantification of terminal protrusions in ddaE neurons, showing that expression of the wild-type (*cathD*^*wt*^) or proteolytically-inactive cathD (*cathD*^*D231N*^) rescues the over-branching phenotype in terminals upon *cathD* depletion (*cathD*^*1*^). **D** Sholl analysis of dendrites of ddaE neurons. Note an increase of intersections at 50–60 μm from the soma in cathD-deficient (*cathD*^*1*^) neurons, which is abolished by reintroduction of wild-type (*cathD*^*wt*^) or proteolytic-inactive (*cathD*^*D231N*^) cathD. **E–G** Quantification of dendrite branch length in primary (**E**), secondary (**F**), and tertiary dendrites (**G**) in ddaE neurons, showing the rescue effects of both wild-type (*cathD*^*wt*^) and proteolytically-inactive (*cathD*^*D231N*^) cathD upon cathD deficiency (*cathD*^*1*^). Data are shown as the mean ± SEM; **P < 0.01, ***P < 0.001 by one-way ANOVA with Tukey’s multiple comparisons test.

A non-proteolytic role of cathD in dendritic morphogenesis was also found in ddaE class I da neurons. Compared with wild-type controls, cathD deletion increased the dendritic complexity of ddaE neurons, with extensive and tangled branches adjacent to the soma, which was rescued upon expression of wild-type or proteolytically-inactive cathD (Fig. 4A). Similar to the phenotype in ddaD neurons (Fig. 2), loss of cathD markedly increased the branch number, terminal protrusions, and dendritic intersections (Fig. 4B–D). Furthermore, cathD depletion increased the branch length in tertiary dendrites, without altering it in primary and secondary dendrites of ddaE neurons (Fig. 4E–G). In addition, wild-type and proteolytically-inactive cathD both rescued these morphological defects (Fig. 4B–G), supporting a non-proteolytic role of cathD in regulating dendritic morphogenesis.

### CathD Controls Mushroom Body Morphogenesis

**Fig. 5:**
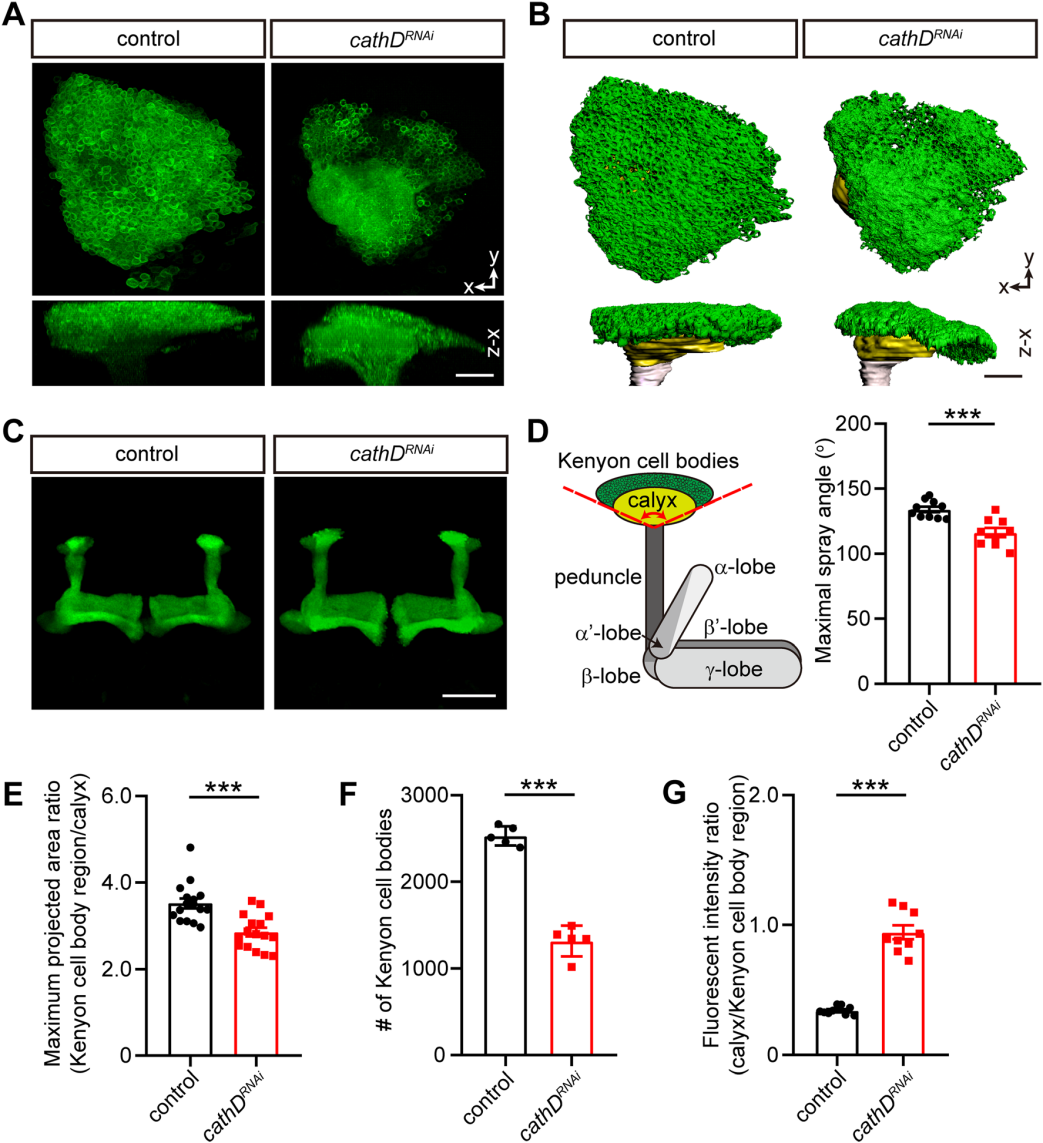
CathD regulates neuronal morphogenesis in *Drosophila* mushroom bodies. **A**, **B** Representative fluorescence (**A**) and 3D reconstruction (**B**) images of *OK107-Gal4; UAS-mCD8-GFP-*labeled mushroom bodies, showing a reduction of Kenyon cells (KCs) (green regions in **B**), and an increase of fluorescent intensity in the calyx (yellow regions in **B**) upon cathD knockdown (*cathD*^*RNAi*^) (scale bars, 20 μm). **C** Representative images of axonal lobes in the mushroom bodies, showing no significant difference between control and cathD knockdown (*cathD*^*RNAi*^) flies. **D** Schematic and quantification of the coverage by the KC somal region atop the calyx. Measured by the maximal spray angles between the margins of the KC somal region (left panel), the coverage was reduced upon cathD knockdown (*cathD*^*RNAi*^). **E** The maximal projected area ratio between the KC somal region and the calyx. **F** Numbers of KCs, showing a decrease upon cathD knockdown (*cathD*^*RNAi*^). **G** Fluorescence intensity ratio between the dendritic region and KC somal region, showing a relative increase upon cathD knockdown (*cathD*^*RNAi*^). Data are shown as the mean ± SEM; ***P < 0.001, unpaired Student’s *t* test or Mann–Whitney *U* test.

We next determined whether cathD regulates neuronal morphogenesis in the CNS of *Drosophila*. Located in the dorsal cortex, MBs are composed of Kenyon cells (KCs) and their processes [21, 22]. Driven by OK107-Gal4, cathD expression was silenced by cathD-specific RNA interference (RNAi) in MBs. Upon cathD knockdown, decreased volume of the cell bodies and increased GFP intensity in the dendritic regions (calyx) were observed in KCs (Fig. 5A, B). Meanwhile, the axonal projections of cathD-knockdown KCs (α/β/γ lobes of MBs) appeared similar to wild-type controls (Fig. 5C), suggestive of a selective regulation by cathD during MB development.

Further analysis revealed that cathD knockdown decreased the coverage of KC somata atop the calyx, accompanied by a decrease in the number of KCs (Fig. 5D–F), suggesting that cathD directly controls neuronal development in the CNS. Moreover, compared with the cell body region, the fluorescence intensity of the calyx was dramatically increased upon cathD knockdown (Fig. 5G), implying an overgrowth of dendritic processes in the MBs. These data demonstrate that cathD plays a crucial role in MB morphogenesis, especially for KCs and their dendrites.

**Fig. 6:**
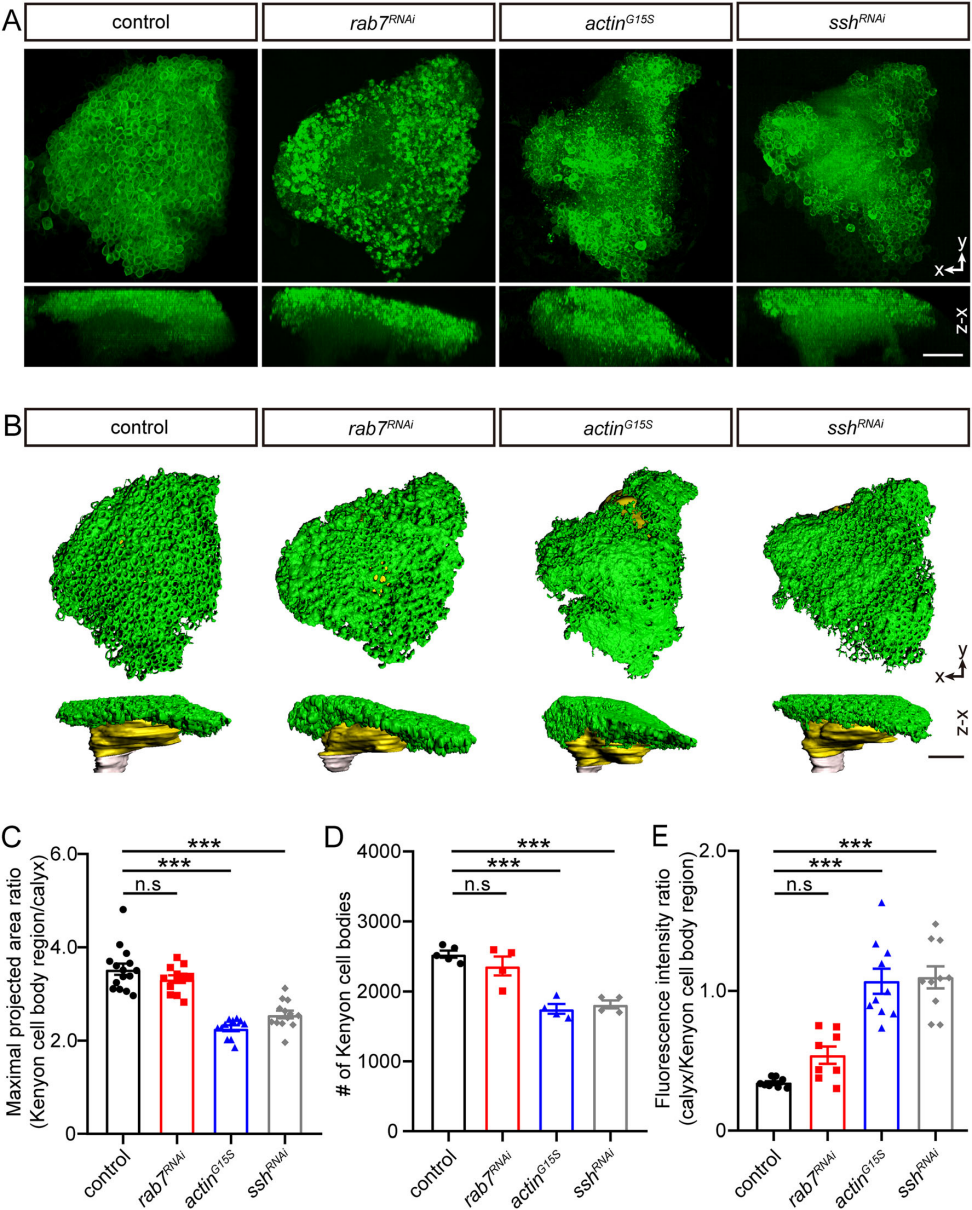
Knockdown of Rab7 does not alter mushroom body morphogenesis. **A**, **B** Representative fluorescence (**A**) and 3D reconstruction (**B**) images of *OK107-Gal4; UAS-mCD8-GFP*-labeled mushroom bodies, showing no morphological changes in Kenyon cells (KCs) (green regions in **B**) and the calyx (yellow regions in **B**) upon Rab7 knockdown (*rab7*^*RNAi*^), whereas actin^G15S^ overexpression (*actin*^*G15S*^) and slingshot knockdown (*ssh*^*RNAi*^) both lead to fewer KCs and increased intensity of calyx staining (scale bars, 20 μm). **C** Ratio of maximal projected area of the KC somal region and the calyx. **D** Numbers of KCs, showing no significant changes upon Rab7 knockdown. **E** Fluorescence intensity ratio of the dendritic region and KC somal region. Data are shown as the mean ± SEM; ***P < 0.001, one-way ANOVA with Tukey’s multiple comparisons test.

Rab7 is a small GTPase that is required for cathD maturation by facilitating lysosome fusion and acidification [23]. We next applied specific Rab7-RNAi to suppress Rab7 expression and inhibit cathD maturation, thus reducing the proteolytic activity of cathD in MBs. Intriguingly, no change was observed in MBs upon knockdown of Rab7 (Fig. 6A, B), raising the possibility that cathD regulates MB morphology independent of its proteolytic activity. Furthermore, overexpression of over-stabilized actin (G15S) and knockdown of cofilin phosphatase slingshot (ssh) both reduced the number of KCs and increased the fluorescent intensity of the calyx (Fig. 6A, B), similar to the phenotype caused by cathD deficiency (Fig. 5). Detailed analysis indicated that overexpression of actin^G15S^ or ssh significantly decreased the coverage of KC somata atop the calyx and the number of KCs, but increased the fluorescence intensity of the calyx (Fig. 6C–E), sharing defects similar to those with *cathD* depletion (Fig. 5). Together, these results suggest that cathD regulates neuron morphology *via* an actin-mediated mechanism.

## Discussion

A highly-conserved protease in the endosomal/lysosomal system, cathD is well characterized for its degradative function. Besides its traditional proteolytic role, emerging evidence suggests that cathD plays non-proteolytic roles in controlling biological processes [2, 4, 24, 25]. In the present study, we demonstrated that cathD non-canonically modulates neuronal morphogenesis in both larval and adult stages. Although the underlying mechanism remains to be investigated, our study sheds light on a non-canonical function of cathD in nervous system development.

Previous studies have reported that the depletion or inactivation of cathD leads to progressive neuronal lipofuscinosis and neurodegeneration, demonstrating that its proteolytic activity is required for maintaining neuronal homeostasis [2, 10–12]. Here, we describe aberrant dendritic features in cathD-deficient *Drosophila*, including over-branching, aberrant turning, and elongation defects (Figs. 1, 2, 3 and 4), providing novel evidence that cathD regulates neuronal morphogenesis. Differing in dendritic process orientation and dendritic field size, both class I and class III neurons require cathD for normal dendritic architecture (Figs. 1 and 2), indicating that cathD plays a general role in controlling sensory neuron morphology.

Like wild-type cathD, proteolytically-inactive cathD^D231N^ efficiently rescued the dendritic abnormalities caused by *cathD* depletion (Figs. 3 and 4), indicating that the regulatory role of cathD in dendritic morphology is independent of its degradative action. Along with previous reports on the non-proteolytic roles of cathD in different biological processes, our results point to a distinct non-proteolytic role of cathD in orchestrating neuronal development. Although further exploration is required to unveil the underlying molecular mechanism, our results show that stabilization of the actin cytoskeleton by the actin^G15S^ mutant results in phenotypes similar to cathD-depleted dendrites (Fig. 3), implying a modulation of actin dynamics by cathD in neurite growth.

Actin dynamics is crucial for neurite turning by controlling the orientation and speed of growth-cone movement [26, 27]. Proteomics and bioinformatics studies have demonstrated that cathD deficiency alters the levels of protein associated with the actin cytoskeleton and the organization of cell projections, and leads to aberrant cell adhesion [28]. Therefore, we speculate that cathD regulates dendrite growth *via* an actin-based process.

Besides sensory neurons in the peripheral nervous system, our results also provide evidence that cathD controls the dendritic arborization of neurons in the CNS. In our observations, cathD knockdown reduced the KC population and promoted dendritic growth in the calyx as shown by dramatically enhanced process intensity in the dendritic region (Fig. 5). In addition, cathD knockdown did not affect the axon region of KCs (Fig. 5). Taken together, our study demonstrates that cathD non-proteolytically regulates neuronal morphogenesis in the central and peripheral nervous systems of *Drosophila*, with a potential preference for the regulation of dendritic morphogenesis.

The contribution of cathD to organ development appears to become increasingly important during evolution. CathD mutant flies display lysosomal storage disorders with a normal lifespan [12], while cathD-deficient mice exhibit rapidly progressive multi-system degeneration and die around postnatal day 24 [29]. In addition, cathD-deficient infants exhibit severe multiple organ dysfunctions at birth, and only survive up to a few days [30]. These findings suggest that cathD has essential functions orchestrating diverse vital processes and is indispensable in mammalian species. We propose that cathD non-proteolytically modulates a wide set of biological processes beyond its traditional role in protein degradation. The underlying mechanisms and other potential functions of cathD, especially during evolution, merit further exploration.

## Electronic supplementary material

Below is the link to the electronic supplementary material.

Supplementary material 1 (PDF 81 kb)
