# Supplementary material for: Lysosomal Hydrolase Cathepsin D Non-proteolytically Modulates Dendritic Morphology in Drosophila
Source: Neurosci Bull. 2020 Mar 14;36(10):1147–57. doi: 10.1007/s12264-020-00479-6 (PMC7532236; doi:10.1007/s12264-020-00479-6)
Supplement: Supplementary file 1 — Supplementary material 1 (PDF 81 kb) [file 12264_2020_479_MOESM1_ESM.pdf]

Table S1. Summary of statistical analysis results

Figure	Statistical method	Statistical results		*P* value
1B	Unpaired Student's *t* test	$t_{26} = 2.893$		<0.01**
1C	Unpaired Student's *t* test	$t_{19} = 5.525$		<0.001***
1E	Unpaired Student's *t* test	$t_{19} = 4.469$		<0.001***
1F	Unpaired Student's *t* test	$t_{19} = 4.827$		<0.001***
2B	Two-way ANOVA	Interaction $F_{(15, 144)} = 3.810$		<0.001***
		Row Factor $F_{(5, 144)} = 151.9$		<0.001***
		Column Factor $F_{(3, 144)} = 23.25$		<0.001***
		2nd branch	$cathD^{1}$ vs. $cathD^{1}$+$cathD^{wt}$	<0.01**
			$cathD^{1}$ vs. $cathD^{1}$+$cathD^{D231N}$	<0.001***
		3rd branch	control vs. $cathD^{1}$	<0.001***
			$cathD^{1}$ vs. $cathD^{1}$+$cathD^{wt}$	<0.001***
			$cathD^{1}$ vs. $cathD^{1}$+$cathD^{D231N}$	<0.001***
		4th branch	control vs. $cathD^{1}$	<0.01**
			$cathD^{1}$ vs. $cathD^{1}$+$cathD^{wt}$	<0.001***
			$cathD^{1}$ vs. $cathD^{1}$+$cathD^{D231N}$	<0.001***
2C	One-way ANOVA	$F_{(3, 42)} = 10.16$		<0.001***
	Tukey's multiple comparisons test	control vs. $cathD^{1}$		<0.01**
		$cathD^{1}$ vs. $cathD^{1}$+$cathD^{wt}$		<0.01**
		$cathD^{1}$ vs. $cathD^{1}$+$cathD^{D231N}$		<0.001***
2D	Two-way ANOVA	Interaction $F_{(81, 554)} = 1.331$		<0.05*
		Row Factor $F_{(27, 554)} = 78.47$		<0.001***
	Tukey's multiple comparisons test	x=50.621	control vs. $cathD^{1}$	<0.01**
		x=60.621	control vs. $cathD^{1}$	<0.05*
2F	One-way ANOVA	$F_{(3, 26)} = 9.311$		<0.001***
	Tukey's multiple comparisons test	control vs. $cathD^{1}$		<0.001***
		$cathD^{1}$ vs. $cathD^{1}$+$cathD^{wt}$		<0.05*
		$cathD^{1}$ vs. $cathD^{1}$+$cathD^{D231N}$		<0.01**
2G	One-way ANOVA	$F_{(3, 26)} = 22.50$		<0.001***
	Tukey's multiple comparisons test	control vs. $cathD^{1}$		<0.01**
		$cathD^{1}$ vs. $cathD^{1}$+$cathD^{wt}$		<0.001***
		$cathD^{1}$ vs. $cathD^{1}$+$cathD^{D231N}$		<0.001***
3B	One-way ANOVA	$F_{(5, 141)} = 17.35$		<0.001***
	Tukey's multiple comparisons test	control vs. $cathD^{1}$		<0.01**
		$cathD^{1}$ vs. $cathD^{1}$+$cathD^{wt}$		<0.001***
		$cathD^{1}$ vs. $cathD^{1}$+$cathD^{D231N}$		<0.001***

			control vs. $actin^{G15S}$	<0.01**
4B	Two-way ANOVA		Interaction $F_{(15, 144)} = 3.810$	<0.001***
			Row Factor $F_{(5, 144)} = 151.9$	<0.001***
			Column Factor $F_{(3, 144)} = 23.25$	<0.001***
	Tukey's multiple comparisons test	3rd branch	control vs. $cathD^{1}$	<0.001***
			$cathD^{1}$ vs. $cathD^{1}$+$cathD^{wt}$	<0.001***
			$cathD^{1}$ vs. $cathD^{1}$+$cathD^{D231N}$	<0.001***
		4th branch	control vs. $cathD^{1}$	<0.01**
			$cathD^{1}$ vs. $cathD^{1}$+$cathD^{wt}$	<0.01**
			$cathD^{1}$ vs. $cathD^{1}$+$cathD^{D231N}$	<0.001***
4C	One-way ANOVA		$F_{(3,46)} = 11.16$	<0.001***
	Tukey's multiple comparisons test		control vs. $cathD^{1}$	<0.01**
			$cathD^{1}$ vs. $cathD^{1}$+$cathD^{wt}$	<0.01**
			$cathD^{1}$ vs. $cathD^{1}$+$cathD^{D231N}$	<0.001***
4D	Two-way ANOVA		Interaction $F_{(72, 500)} = 2.180$	<0.001***
			Row Factor $F_{(24, 500)} = 105.3$	<0.001***
			Column Factor $F_{(3, 500)} = 26.45$	<0.001***
	Tukey's multiple comparisons test	x=60.621	control vs. $cathD^{1}$	<0.05*
4G	One-way ANOVA		$F_{(3, 23)} = 12.80$	<0.001***
	Tukey's multiple comparisons test		control vs. $cathD^{1}$	<0.01**
			$cathD^{1}$ vs. $cathD^{1}$+$cathD^{wt}$	<0.001***
			$cathD^{1}$ vs. $cathD^{1}$+$cathD^{D231N}$	<0.01**
5D	Unpaired Student's *t* test		$t_{19} = 4.436$	<0.001***
5E	Unpaired Student's *t* test		$t_{30} = 4.393$	<0.001***
5F	Unpaired Student's *t* test		$t_{8} = 12.95$	<0.001***
5G	Mann Whitney U test		U = 0	<0.001***
6C	One-way ANOVA		$F_{(3, 47)} = 38.95$	<0.001***
	Tukey's multiple comparisons test		control vs. $actin^{G15S}$	<0.001***
			control vs. ssh^{RNAi}	<0.001***
6D	One-way ANOVA		$F_{(3, 13)} = 23.26$	<0.001***
	Tukey's multiple comparisons test		control vs. $actin^{G15S}$	<0.001***
			control vs. ssh^{RNAi}	<0.001***
6E	One-way ANOVA		$F_{(3, 33)} = 29.80$	<0.001***
	Tukey's multiple comparisons test		control vs. $actin^{G15S}$	<0.001***
			control vs. ssh^{RNAi}	<0.001***
